# Drivers of phenotypic variation in cartilage: Circadian clock genes

**DOI:** 10.1111/jcmm.16768

**Published:** 2021-07-02

**Authors:** Xiaopeng Song, Hui Bai, Xinghua Meng, Jianhua Xiao, Li Gao

**Affiliations:** ^1^ College of Veterinary Medicine Heilongjiang Key Laboratory Animals and Comparative Medicine Northeast Agricultural University Harbin China

**Keywords:** cartilage, circadian clock, gene, osteoarthritis, phenotype

## Abstract

Endogenous homeostasis and peripheral tissue metabolism are disrupted by irregular fluctuations in activation, movement, feeding and temperature, which can accelerate negative biological processes and lead to immune reactions, such as rheumatoid arthritis (RA) and osteoarthritis (OA). This review summarizes abnormal phenotypes in articular joint components such as cartilage, bone and the synovium, attributed to the deletion or overexpression of clock genes in cartilage or chondrocytes. Understanding the functional mechanisms of different genes, the differentiation of mouse phenotypes and the prevention of joint ageing and disease will facilitate future research.

## 1 INTRODUCTION

**FIGURE 1:**
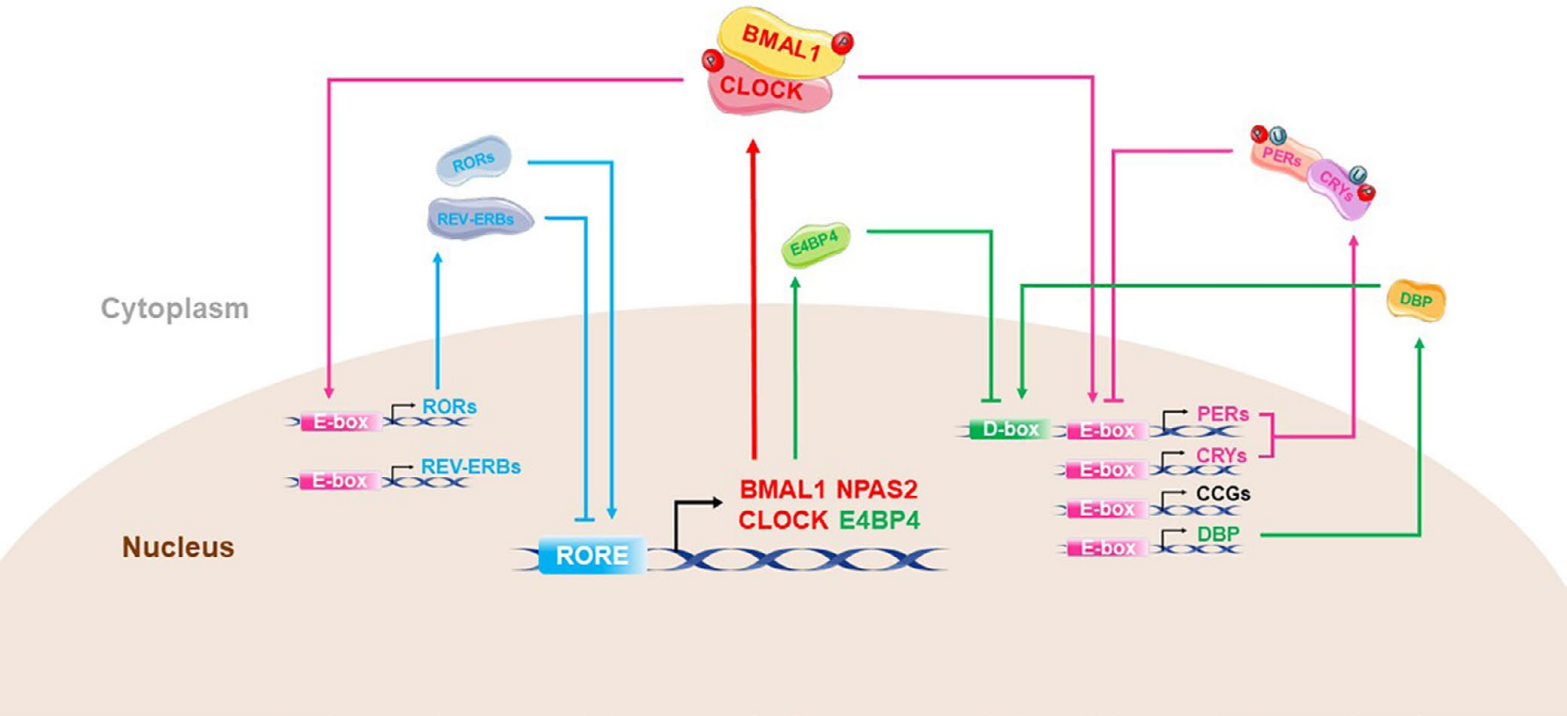
Circadian clock transcription‐translation feedback loops. In primary TTFL, phosphorylated BMAL1:CLOCK/NPAS2 is translocated into the nucleus and initiates the transcription of related genes by binding to the promoter regions E‐box (5′‐CACGTG‐3′) of several genes, including the core clock genes Cryptochrome (CRYs), Period (PERs), REV‐ERBs and retinoic acid receptor‐related orphan receptors (RORs). The PER and CRY proteins accumulate in the cytoplasm, enter the nucleus and inhibit the transcription and phosphorylation of BMAL1 and CLOCK. Consequently, further production of clock proteins in inhibited. In another feedback pathway, RORs and REV‐ERBs proteins competitively bind to ROR elements (ROREs) near the BMAL1 promoter. While REV‐ERBs inhibit the transcription of BMAL1, RORs promote the process. As such, REV‐ERBs show a strong circadian rhythm that is aligned with the rhythm of BMAL1. Additionally, D‐box binding protein (DBP), regulated by E‐box and the mammalian transcription factor E4 binding protein 4 (E4BP4; regulated by ROREs), accommodate the expression of PER gene through D‐box and, in turn, affect E‐box activity.^6^ Overall, these three cis‐acting elements coordinate a circadian cycle over approximately 24 h

Osteoarthritis (OA) is a highly prevalent rheumatic musculoskeletal disorder, encompassing progressive, inflammatory and immunological changes affecting joint structures.^1^ The clinical features of OA include cartilage loss, increased subchondral bone thickness, tidemark replication, decreased subchondral trabecular bone mass and bone marrow lesions (BML).^2^, ^3^ Endogenous biological clocks determine daily, monthly or annual rhythms in biological processes. Cells with endogenous self‐excited oscillations, caused by molecular fluctuations produced by a series of clock genes, form the basis of circadian rhythms. The classical biological clock pathway is comprised of three transcription‐translation feedback loops (TTFL) in mammals (Figure 1). A heterodimer is the core element of TTFLs, which is formed by BMAL1 (brain and muscle ARNT‐like 1/Arntl) and NPAS2 (Neuronal PAS domain protein 2) and/or CLOCK (Circadian locomotor output cycles kaput).^4^, ^5^ Additional proteins in the loops such as Cryptochrome (CRYs), Period (PERs), REV‐ERBs, retinoic acid receptor‐related orphan receptors (RORs) and D‐box binding protein (DBP) are synthesized at specific times of the day, accumulate and degrade in the cytoplasm.

A vast array of studies has elucidated specific genes or external factors that disrupt the body's natural clock. The systemic or specific deletion of classic clock genes in the biological clock's TTFLs and the artificial disruption of the sleep‐wake cycle, causes significant alterations in the human body, including changes in body mass and composition, impaired mental status, growth retardation, premature ageing and cardiovascular disease.^7^, ^8^, ^9^, ^10^ In this review, we summarize the integral articular phenotypic variations and the disordered expression of molecular oscillations caused by the disruption of representative clock genes throughout the whole body or within cartilage. These insights provide valuable clues regarding the biological clock's involvement in maintaining cartilage and joint developmental, ageing, and metabolic processes, and healthy matrix homeostasis.

## 2 EVIDENCE OF CIRCADIAN CLOCK IN CARTILAGE

The circadian rhythm of peripheral tissues can be regulated by specific inputs from external factors, such as eating, temperature, light and physical exercise. These factors provide coordinated input to circadian rhythmicity through crosstalk between metabolic process and neural circuits.^11^, ^12^, ^13^ In 1962, Simmons described diurnal variations in cell proliferation and bone growth plate metabolism.^14^, ^15^ Soon after, the metabolic rhythms of the epiphysis and condylar cartilage were also studied in detail.^16^, ^17^, ^18^, ^19^, ^20^, ^21^, ^22^ Additionally, research reported that, in the absence of alterations in environmental factors, the thickness and volume of articular cartilage also vary from day to night.^23^, ^24^ Collectively, these findings suggest the possibility of an autonomously controlled clock in cartilage. A recent series of PER2::Luc clock reporter mouse model studies more directly demonstrated the autonomic functioning of the circadian clock in cartilage and cartilage explants in vivo.^25^, ^26^, ^27^ With the emergence of DNA microarrays, mass spectrometry and other advanced molecular biological technologies, more clock genes have been detected in cartilage. More than 600 genes and 145 proteins expressed in cartilage have periodic rhythmicity controlled by clock genes.^28^, ^29^, ^30^, ^31^

## 3 CARTILAGE AND BONE PHENOTYPES OF MOUSE CLOCK GENE MUTATIONS

### 3.1 BMAL1

#### 3.1.1 BMAL1 may not be necessary for embryonic limb cartilage development and bone growth

Many studies have shown that *BMAL1* deficiency significantly reduces early embryonic development and the implantation potential of female mice.^32^, ^33^, ^34^ However, in normal mouse embryo development, BMAL1 might not be required for cartilage and long bone development. Yu et al using Alcian blue and Alizarin red staining analysis found insufficient cranial cartilage calcification and smaller and shorter mandibular condyle phenotypes in E14.5‐E18.5 embryos of global *BMAL1* deletion and *BMAL1^fl/fl^ Twist‐Cre* mice.^35^ This finding suggests that BMAL1 occupies a crucial position in the chondrogenesis and entochondrostosis processes of the mandibular condyles. However, the abnormal progression of endochondral ossification in the limbs was not mentioned. To further understand its role, Ma et al specifically ablated *BMAL1* from the cartilage of E13.5‐E18 mice. They found no abnormalities in body mass, bone length (femur, tibia and humerus) and growth plates compared to wide‐type (WT) mice,^36^ suggesting that BMAL1 is not necessary for the elongation of long bones (Table 1). The sharp contrast between the two studies may be attributed to the different stages of endochondral osteogenesis between the mandibular condyle and limb bones.

**TABLE 1:** Summary of phenotypes of genome‐editing mouse models in cartilage circadian clock disruption. Description of mouse models in the foetus, adolescence and adult stage in terms of alterations of body height/weight, cartilage/growth plate and bone compared with age‐matched wide‐type mice

References	Mice	Detection of the *Δ*allele	Age	General	Location	Visual observation and pathological		Protein
						Cartilage tissue/growth plate	Bone and other tissues	Cartilage tissue
*Foetus stage*								
Yu, et al^35^	***Bmal1*** *^−/−^*	Exon 5 and part of exon 4	E14.5‐18.5	Body size decreased	Mandibular condyle	Chondrogenesis decreased	Alizarin red staining decreased	COL2A1, COL10A1, ACAN, SOX9, CyclinD1 and Ki67 decreased; TUNEL‐positive cells increased
	***Bmal1^fl/fl^; Twist2‐Cre***							
Ma, et al^36^	***^TamCart^ Bmal1^fl/fl^***	Not mentioned	E13.5‐18.5	No abnormal				
Adolescence stage								
Bunger,MK., et al ^46^	***Bmal1*** *^−/−^*	Exon 5 and part of exon 4	W6‐11	No abnormal				
Yu, et al ^35^	***Bmal1*** *^−/−^*	Exon 5 and part of exon 4	W4‐20	——	Mandibular condyle	Shorter PZ and HZ; number of chondrocytes per column in PZ and HZ decreased	BMD, BV/TV, Tb. Th and Tb.N decreased; Tb. Sp increased	COL2A1, ACAN, COL10A1 and Ki67 decreased; TUNEL‐positive cells increased
Suyama,K., et al ^47^	***Bmal1*** *^−/−^*	Exon 5 and part of exon 4	W10	——	Lumbar spine	Disc height and DHI decreased; lower matrix‐to‐cell ratio in NP; hyperplasia in AF	Vertebral bone BV, TV, BV/TV, Tb.N and Tb. Th decreased; Tb. Sp increased	——
Ma, et al ^36^	***^TamCart^ Bmal1^fl/fl^***	Not mentioned	W4‐6	Body weight and length decreased	Hindlimb	Shorter PZ; larger HZ	Shorter	BrdU‐positive cells, COL10A1, VEGF, HIF1α, and Bcl‐2 decreased; TUNEL‐positive cells, Caspase‐3, MMP13 and Runx2 increased
Dudek, et al ^44^	***Col2α1‐Bmal1*** *^−/−^*	Exon 8	W4‐12	No abnormal	Knee joint	Cell apoptosis near the tidemark; loss of chondrocytes and voids in ECM	No abnormal	——
Takarada, et al ^45^	***Bmal1*** *^−/−^*	Exon 6‐8	W3‐15	Body length/weight decreased	Rib	——	——	IHH decreased; no abnormal with Col II, Col X, Sox9, Runx2
	***α1(II)‐collagen‐Cre; Bmal1^fl/fl^***				Hindlimb	——	Shorter	IHH decreased
*Adult stage*								
Bunger, et al^46^	***Bmal1*** *^−/−^*	Exon 5 and part of exon 4	W20‐40	Body weight decreased; abnormal gait and posture	Tarsal joint	——	Calcified nodules; calcaneal tendon calcification; osteocalcin increased; no abnormal in BMD	——
					Vertebrae	Proliferative bone bridging from the costal cartilage to the sternum; increased matrix deposition and bone proliferation in perichondrium	Bridging ankylosis and osteophyte in intervertebral and lumbar joints; no abnormal in BMD	——
					Knee joint	No abnormal	Tendons and ligaments ectopic calcification/ossification; no abnormal in BMD	——
Schroder, et al ^54^	***Bmal1*** *^−/−^*	Exon 8	W20‐22	——	Hindlimb and Ribcage	Alcian Blue decreased	Distal tibia thicker; calcification of the calcaneal tendon	——
	**iMS‐*Bmal1*** *^−/−^*		W75‐79	Hair loss; dermatitis increased; kyphosis				
Dudek, et al^52^	***Col2α1‐Bmal1*** *^−/−^*	Exon 8	W24‐48	——	Intervertebral disc	Thinner; gradual disappearance of CEP; bone bridges; fibrosis	Ectopic ossification	Adamts1, Adamts5, Adamts15 and Follistatin increased
Hand, et al^56^	**Col6α1‐*Bmal1*** *^−/−^*	Exon 8	W12‐36	——	Ankle joint	——	Density increased; calcaneal spur; calcification between the metatarsal and phalangeal bones; chondroid metaplasia at the junction of the synovium, joint capsule and enthesis repair tissue	——
					Intervertebral disc	Calcification	——	——
Yuan, et al ^58^	***Clock^Δ19^***	Exon 19	W24	Knee joint	——	Safranin‐O staining decreased; cartilage and meniscus damage histologic scores increased	——	——
Kc, et al ^11^	***Clock^Δ19/Δ19^***	Exon 19	W7‐9	Knee, shoulder, spine disc and facet joints	No abnormal			

Note

PZ: Proliferative zone; HZ: Hypertrophic zone; BMD: Bone mineral density; BV/TV: bone volume/total volume; Tb.Th: Trabecular thickness; Tb.N: Trabecular number; Tb.Sp: Trabecular separation; CEP: Cartilaginous end plate; IVD: Intervertebral disc; DHI: Disc height index; NP: Nucleus pulposus; AF: Annulus fibrosus; ECM: Extracellular matrix.

The primary synovial joint articular cartilage grows from chondrocytes in the central layer of the epiphyseal plate, gradually differentiated from E11.5. Its shape and biosynthetic properties remain invariable throughout its lifetime.^37^, ^38^ Inversely, condyle cartilage, as secondary cartilage, does not develop significantly until E15. Growth starts from mesenchymal tissue covering the prenatal or postnatal condyle, and it easily adapts to alterations in the environment.^39^, ^40^ In contrast to the significant effect of BMAL1 on the postnatal endochondral ossification of bone (detailed below), studies have shown that these gene products may not yet form a functional circadian feedback loop during the early stages of development.^41^, ^42^, ^43^ However, more research is needed to support this proposal.

#### 3.1.2 BMAL1 significantly affects adolescent cartilage and bone development

Although the expression of BMAL1 in cartilage and bone gradually decreases with body development, its inverse role is indispensable.^44^ Genome‐wide RNA sequencing shows the differential expression of chondrogenesis‐related genes, and Hedgehog pathway‐related proteins were highest during adolescence (4‐6 weeks) compared to postpubescence (8‐10 weeks).^35^ BMAL1 and CLOCK were highly expressed by proliferous zone (PZ) or prehypertrophic to hypertrophic zone (HZ) chondrocytes in the growth plates of WT mice,^35^, ^45^ which alluded that BMAL1 was closely related to chondrogenesis.

The study of mice with global *BMAL1* deletion was pioneered by Bunger et al.^46^ No significant abnormal changes were observed in body mass, body length, degenerated bone and joint pathology before 15 weeks of age in *MOP3^−/−^* mice. Alternatively, Bunger et al suggested that MOP3 was critical for maintaining joint homeostasis in adulthood. In another study, body mass and length, as well as the longitudinal length of the tibia and femur, of *BMAL1^−/−^* mice were significantly reduced from three weeks of age, indicating that BMAL1 influences the elongation of long bones, but it does not indicate obvious cartilage pathology.^45^ However, in a study of the mandibular condyle, Yu et al ^35^ reported that cartilage thickness and matrix were significantly reduced across the whole‐body *BMAL1*‐deleted mice, accompanied by reduced chondrocyte proliferation and increased apoptosis. These findings are completely contrary to the previous conclusions, possibly due to the reasons proposed above. Using microcomputed tomography to evaluate intervertebral disc height in mice, the annulus fibrosus (AF) tissue of *BMAL1^−/−^* mice was shown to be hyperplasic, the lumbar vertebrae and intervertebral discs were significantly smaller and thinner, and the vertebral bone parameters were also significantly reduced.^47^ A possible explanation for the distinct cartilage pathology at these different anatomical sites may be that articular cartilage maintains a low‐level of metabolic synthesis and proliferation after birth. In contrast, secondary cartilage or intervertebral discs are sensitive to external stimuli and mechanical pressures and can deform flexibly.

Several transgenic mice variants with specific *BMAL1* deletion from the cartilage have been constructed to investigate its effect on cartilage homeostasis. Dudek et al established adult chondro‐specific *BMAL1*‐KO mice^44^ and discovered significant degeneration of knee cartilage, extracellular matrix (ECM), cell damage, and tidemark loss, commencing from the eighth week; the observations were restricted to articular cartilage and did not include the synovium or its affiliated ligaments. However, no distinct decreases in body mass and length were observed, which was inconsistent with another study of *αⅠ(Ⅱ)‐collagen‐Cre; Bmal1^fl/fl^* mice.^45^ The conspicuous discrepancy may be explained by differences in the type II collagen a1 (COL2A1) promoter targeting strategies of the two groups of *COL2A1*‐CRE transgenic deletion strains.^48^ Ma et al also reported improvements in skeletal lengths ^36^ and suggested the increase in apoptotic cells, with reductions of collagen (COL), SRY‐Box transcription factor 9 (SOX9), vascular endothelial growth factor (VEGF), other basic components of the cartilage matrix and development related proteins, results in the disappearance of the circadian rhythm. These observations revealed that the deletion of *BMAL1* hindered the mineralization and calcification of normal hypertrophic chondrocytes and delayed angiogenic‐osteogenic coupling. These outcomes appear to be closely linked with the precise regulation of the Indian hedgehog (IHH) protein, a pivotal member of the developmental regulatory Hedgehog family.^35^, ^45^ The BMAL1:CLOCK heterodimer complex could bind to the E‐box (CACGTG) in the promoter region of IHH and hedgehog ligand 1, binding to Patched (PTCH1), a key receptor of hedgehog pathway. This would regulate the sequential differentiation of chondrocytes, which could be influenced by PER1 and parathyroid hormone (PTH).^45^ Early in the mouse lifecycle, the hedgehog pathway activator, SAG, partially saves the mandible shortness phenotype caused by *BMAL1* deficiency rather than in adulthood.^35^ This is consistent with the rate of cartilage repair during development being much higher in the young compared to adults and the elderly, suggesting the reversibility of cartilage formation. Transforming growth factor‐β (TGF‐β) and the nuclear factor of activated T cells (NFAT) signalling also play a non‐negligible role in maintaining stable growth and ageing of articular cartilage ^49^, ^50^ by RNA sequencing in cKO mice. Consistently, TGF‐β signalling has also been significantly altered by an in vitro RNA‐seq of knock‐down *BMAL1* and *NR1D1*.^51^

#### 3.1.3 Lack of BMAL1 accelerates ageing and inflammation of limbs and joints in adult mice

Internal body factors, such as ageing, alter the circadian rhythm in cartilage manifested by classical CLOCK, BMAL1, NR1D1 expression, causing changes in amplitude inversely proportional to age (rather than other clock proteins).^25^, ^35^, ^44^, ^51^, ^52^ As mentioned above, Bunger et al found that after twenty weeks of age, *Mop3^−/−^* mice showed abnormal phenotypes, including reduced body mass, anorexia, dehydration and abnormal gait.^46^ Typical non‐inflammatory joint diseases were found through radiological and histological analysis, and there was enhanced heterotopic ossification of hind limb joints, but there were no significant changes in global bone density. Inversely, the articular cartilage of the tibia and femur, and other soft structures (eg muscles), seemed to remain intact and uneroded. Similarly, degeneration of the lumbar intervertebral disc (IVD) was observed in *Col2a1‐BMAL1* cKO mice after six months, and extensive denaturation, ecstatic ossification and increased fibrosis at twelve months.^52^ These outcomes suggest that BMAL1 significantly disturbs bone homeostasis during the ageing process.

Consistent with previous research,^53^ phenotypic changes in inducible skeletal muscle‐specific deletion of *BMAL1* in adult mice aged 16‐20 weeks were not limited to the muscle transition to an ageing phenotype.^54^ They reported some phenotypes similar to those demonstrated by Bunger et al,^46^ including a limited range of movement, spinal and tail deformity, and increased tendon and ligament calcifications. Complementarily, bone calcification and decreased cartilage staining, similar to the OA‐like joint phenotypes induced by systemic *BMAL1*‐KO mice, were also found in this study. An additional viewpoint was thus proposed that skeletal muscle systems might share a set of peripheral clock regulators enabling synchronization.^55^ Dudek et al provided further evidence for this hypothesis by comparing the rhythm gene sequence of IVD, cartilage and tendon and identified eight core clock genes (including *ARTNL*, *PER2* and *Dbp*) among the rare number of overlapping genes.^52^

By ablating *BMAL1* from articular mesenchymal cells expressing *COL6A1*,^56^ overall joint alterations further encompassed changes in fibroblast‐like synoviocytes (FLS) and MHCⅡ macrophages in the synovium, owing to FLS and chondrocytes originated from the progenitor cells. Similar to Bunger et al, significant thickening of the ankle joint synovium, cartilage and bone hyperplasia and chondroid metaplasia were interspersed at the tendon and ligament insertion sites.^46^ However, no further variations in the knee joint were recorded. These phenotypes provide additional evidence of the biological clock's central role in maintaining healthy joints, as the FLS can secrete a myriad of inflammatory factors leading to RA. Likewise, the synovial membrane is the only tissue in the joint cavity filled with blood vessels. As such, inflammatory factors may damage adjacent tissues through the circulatory system.

### 3.2 Other clock genes

As another pivotal member of the BMAL1:CLOCK heterodimer, CLOCK also plays a noteworthy role in maintaining normal articular cartilage. Large‐scale DNA microarray analysis showed that CLOCK might be a mechanically sensitive clock gene, as it was significantly up‐regulated following mechanical stress.^30^ According to Safranin‐O staining, abnormal weight loss and cartilage degeneration phenotypes, similar to *BMAL1* deletion, were found in *CLOCK^Δ19^* mice, where the nuclear factor κB (NF‐κB) signalling was activated to trigger chronic inflammation.^57^, ^58^ This was inconsistent with the vacant study of Kc et al,^11^ who found no significant pathological differences in Safranin‐O fast green staining in the cartilage of *CLOCK* and *Tau*‐mutant mice. This might be due to age differences, as the genetic background was the same between the studies. The loss of *CLOCK* gene fragments may alter cartilage phenotypes in adult mice. In addition to cartilage degradation, shorter tibiae and lower bone mass could also be induced by *CLOCK* mutation.^59^ The latest research demonstrated that the motor ability of 18‐month‐old mice, promoted by injecting lentiviral vectors encoding *CLOCK*, to enhance cartilage regeneration, decreased ageing markers (*Cdkn1a* and *Cdkn2a*), as well as activating genes involved in cartilage development.^60^ These results imply that the *CLOCK* gene supports cartilage rejuvenation and alleviates ageing. However, the importance of other clock genes, such as *CRYs* and *PERs*, in maintaining joint phenotypes has not been studied.

## 4 MOLECULAR EXPRESSION DISORDER CAUSED BY DELETION OF SPECIFIC CLOCK GENES IN CARTILAGE AND CHONDROCYTES

### 4.1 BMAL1

As a core clock factor in the circadian rhythm feedback loop, BMAL1 is crucial for governing the prosodic expression of other rhythm genes and internal or external destructing factors influencing cartilage. RNA‐seq and PER2::Luc bioluminescence imaging confirmed the loss of circadian rhythm in the cartilage of *BMAL1*‐KO/cKO mice.^44^, ^52^, ^56^ Systemic or cartilage‐specific elimination of *BMAL1* impaired the circadian rhythm of postpartum mice, showing significant loss of prosodic expression of several genes associated with cartilage, along with a distinct elevation in the expression of Matrix metalloproteinase 3 (MMP3), MMP13, VEGF and Runt‐related transcription factor 2 (Runx2).^9^, ^36^ Of note, BMAL1 was expressed irregularly in chondrocytes cultured in normal and *BMAL1*‐cKO mice ^36^ compared to other studies.^28^, ^51^ These two opposing manifestations may be due to a discrepancy in the way the chondrocytes were treated or because different species of organisms were used. *siBMAL1* enhanced the reduction of proteoglycan and COL2A1 caused by IL‐1β and increased chondrogenic degrading enzymes,^61^ which was consistent with the trends observed in *BMAL1^−/−^* mouse chondrocytes.^45^ Meanwhile, Sirt1, NAD, and NAMPT were significantly dampened by knocking down *BMAL1* in light of the combination of BMAL1 and Sirt1,^61^ providing an effective basis for the disorders of circadian rhythm seen with ageing.

A significant decrease in PER1 and REV‐ERBα expression was observed in *Bmal1^−/−^* mice, or chondrocytes and FLS, with elevated levels of PER2 and CRY1, but not CRY2.^36^, ^45^, ^56^ BMAL1 and PER2/CRY1 were consistently expressed inversely,^62^ evidenced by robust oscillations in the expression of BMAL1::Luc reporter in SW‐1353 over several days, in antiphase to PER2::Luc reporter.^25^, ^29^ Remarkably, *BMAL1* excision deleted both *BMAL1* and *BMAL2*, which could not substitute *BMAL1* to maintain the expression oscillation of most genes in the tissues.^63^ These results allude to the consistent regulatory role of *BMAL1* on other clock genes. In contrast, other studies have shown that BMAL1 was incapable of mediating RORA, RORC and RBX1.^51^

### 4.2 *Other* *clock genes*

#### 4.2.1 CLOCK

There have been no obvious rhythmic fluctuations in the *CLOCK* gene detected in mouse cartilage tissues, such as rib growth plates ^45^ and intervertebral discs.^64^ It is possible that NPAS2 is combining with BMAL1 instead of the non‐oscillating CLOCK. The oscillations of protein expression in xiphoid tissue cultures disappeared after *CLOCK* mutation,^25^ leading to the nuclear translocation of NF‐κB, acetylation inhibition and phosphorylation activation.^58^ These observations shed light on the role clock genes play in the probability of developing OA.

#### 4.2.2 PERs

Overexpressing *PER1* attenuates ALP, COLⅡ, COLⅩ, SOX6 and IHH expression in ATDC5 cell lines,^45^, ^65^ which are pivotal to chondrogenesis. Thus, PER1 could inhibit the biological processes of chondrocyte differentiation to osteoblasts or hypertrophic chondrocytes. Of note, *PER1* was not sensitive to these genes in primary costal chondrocytes,^45^ which explains the diverse roles of clock genes in the different stages of cartilage formation. Rong et al found that *SOX9* reduction and overexpression of chondrodegradation‐related genes *MMP13* and *ADAMTS5* caused by IL‐1β (but not *BMAL1*) were significantly neutralized by *PER2* knock‐down in human chondrocytes.^66^ This reflects the irreplaceability of BMAL1 in the biological clock pathway.

#### 4.2.3 REV‐ERBs

Although they are members of the secondary circadian clock pathway feedback loop, REV‐ERBs in chondrocytes are infrequently studied. After suppressing *REV‐ERBα* in chondrocytes, Yang et al demonstrated the significant activation of BMAL1 and Sirt1, produces a similar inflammation‐neutralizing effect as the overexpression of *BMAL1* (mentioned above).^61^ As such, the reduction of *REV‐ERBα* results in the retention of BMAL1 in the cytoplasm in the clock pathway feedback loop. However, in the treatment of specific clock genes, drugs targeted at REV‐ERBs and RORs have shown remarkable efficacy against diabetes, atherosclerosis, autoimmune diseases and cancer.^67^ Das et al confirmed that *REV‐ERB* agonist SR9009 alleviates cartilage matrix loss due to OA surgery and significantly reduces pain through intra‐articular injection compared with partial medial meniscectomy (PMM) after 12 weeks of drug administration.^68^ In addition, SR9009 and CRY1/2 agonist KL001 reduce abnormal collagen fibre synthesis and collagen accumulation.^69^ These findings have proved the importance of the circadian rhythm in balancing collagen fibre synthesis and degradation.

## 5 FUTURE RESEARCH DIRECTIONS

Currently, most clock genes are involved in maintaining cartilage health, yet several new challenges have emerged that require further research. First of all, a limitation of the experimental identification and verification methods to elucidate gene function exists; the pathological phenotypes of model mice cannot fully reflect the variations of in vivo function. This is because the level of aspartate aminotransferase (AST), alanine transaminase (ALT) and urea nitrogen (BUN) in the blood of *MOP3^−/−^* mice aged about four months was significantly higher than that of their *MOP3^+/‐^* or *MOP3^+/+^* siblings. In contrast, no remarkable pathological changes were observed in the livers, hearts, lungs and kidneys.^70^ Therefore, further studies into the cellular and molecular mechanisms of temporal and spatial gene expression profiles, along with predictions of their functions, need to be explored. Additionally, the biological clock has cellular specificity. Many researchers have proposed variant ways (differing between cells) in clock genes interact with other genes.^36^, ^47^, ^71^ Furthermore, recent research has identified that some genes expressed in periodic 24‐27 h oscillations after synchronization in isolated liver and skin tissues of *BMAL1^−/−^* might be due to ETS transcription factor and PRDX protein.^63^ These observations highlight the complex and sophisticated regulatory role of BMAL1 function in transcription, post‐transcription, translation and post‐translation of protein expression in different tissues. But it has not yet been reported in cartilage. Our subsequent work will focus on the decisive role of specific core clock genes in inflammatory or degenerative arthropathies or in combination with other proteins to maintain homeostasis in healthy cartilage.

## CONFLICT OF INTEREST

The authors confirm that there are no conflicts of interest.

## AUTHOR CONTRIBUTIONS

**Xiaopeng Song:** Conceptualization (lead); Investigation (lead); Resources (lead); Writing‐original draft (lead); Writing‐review & editing (lead). **Hui Bai:** Formal analysis (equal); Resources (equal); Writing‐review & editing (equal). **Xinghua Meng:** Software (lead); Writing‐review & editing (equal). **Jianhua Xiao:** Supervision (equal); Validation (equal). **Li Gao:** Funding acquisition (lead); Supervision (lead); Validation (lead).

## Data Availability

Data sharing is not applicable to this article.
